# Current status and influencing factors of social support for main caregivers of children with traumatic brain injury: a cross-sectional survey

**DOI:** 10.1186/s13052-025-01906-y

**Published:** 2025-03-26

**Authors:** Deli Liu, Minghui Hu, Li Ma, Qing Yan, Yiming Liu, Ping Li

**Affiliations:** 1 Department of Neurosurgery, Children’s Hospital of Nanjing Medical University, Nanjing, China; 2 Department of Outpatient, Children’s Hospital of Nanjing Medical University, Nanjing, China

**Keywords:** Social support, Caregivers, Children, Traumatic brain injury, Care, Nursing

## Abstract

**Background:**

Children with Traumatic Brain Injury (TBI) frequently undergo rapid fluctuations in their health status, imposing significant strain on their caregivers. This study aimed to evaluate the social support available to primary caregivers of pediatric TBI patients, to provide actionable insights to improve clinical support systems.

**Methods:**

Primary caregivers of pediatric TBI patients treated at our hospital from February 1, 2024, to August 31, 2024, were included in this study. The Social Support Rating Scale was utilized to assess the social support of these caregivers. Pearson correlation analysis and logistic regression were conducted to identify determinants significantly associated with the level of social support.

**Results:**

Two hundred forty-two primary caregivers of children with TBI were enrolled in this study. The mean social support score for these caregivers was 38.52 (± 9.29), suggesting a moderate level of social support. Correlations were identified between social support scores and several caregiver characteristics: age (*r*=-0.564), educational level (*r* = 0.602), place of residence (*r* = 0.542), and monthly per capita family income (*r* = 0.633). Logistic regression analysis revealed that age (OR = 2.184, 95%CI: 1.904–3.022), educational level (OR = 2.462, 95%CI: 1.755–4.086), residence (OR = 2.189, 95%CI: 1.805–2.753), and monthly per capita family income (OR = 3.428, 95%CI: 2.402–4.216) significantly influenced the level of social support.

**Conclusion:**

Enhancing the social support for primary caregivers of children with TBI is imperative. Clinical healthcare providers are advised to develop and implement targeted interventions aimed at the modifiable factors identified, in order to bolster the social support levels of these caregivers.

## Introduction

Globally, over 50 million individuals suffer from traumatic brain injury (TBI) annually, with a disproportionately high incidence rate among children and adolescents under the age of 18 [1]. Data from a study in the United States reveal that out of 5,429 TBI-related hospital visits, 3,971 patients were under 20 years of age [2]. The elevated incidence of TBI in children, coupled with its acute and chronic sequelae, poses significant challenges to society and the economy [3]. The abrupt onset of TBI and the difficulty that affected children and their families face in recognizing signs of disease progression, contribute to an increased psychosocial burden on primary caregivers [4]. This burden not only affects their quality of life but can also lead to depression and other negative emotional states. Moreover, primary caregivers are confronted with substantial financial stress. The process from acute medical intervention to ongoing concerns about disease exacerbation can lead to emotional distress and instability among caregivers, with long-term caregiving potentially imposing a mental health burden [5]. As the most critical component of the social support system for children with TBI, the mental health of primary caregivers not only impacts their own physical and mental well-being but can also influence the children’s enthusiasm and cooperation in treatment [6, 7]. However, in the current healthcare paradigm, medical professionals often focus predominantly on the patients, with comparatively less attention given to the main caregivers.

Social support refers to the assistance provided by individuals or groups on material or psychological levels. This type of support plays a crucial role in alleviating the stress and burden experienced by primary caregivers [8]. Previous research [9] has indicated that social support can mitigate the negative emotions of primary caregivers through specific mechanisms. The availability of social support is directly related to the weight of caregiving burdens, and interventions that enhance social support for primary caregivers can significantly reduce their caregiving burden [10, 11]. Social support is a key resource for coping with stress and can modulate the impact of stress on individuals. When individuals receive ample social support, their adverse reactions to stressful events are reduced [12]. However, there is currently a lack of clarity regarding the level and status of social support for primary caregivers of children with TBI. In light of this, the purpose of this study is to analyze the current status of social support for main caregivers of children with TBI and how this support affects their caregiving burden. Through this research, we aim to provide reliable evidence to support clinical treatment and care for children with TBI, thereby improving the caregiving experience and quality of life for main caregivers.

## Methods

This study employs a cross-sectional design, and the research protocol has been approved by the Children’s Hospital of Nanjing Medical University (approval number: 202412011-1). Prior to the commencement of the study, written informed consent was obtained from all main caregivers of pediatric patients with TBI who were included in the research. To safeguard the privacy of the participants, the collected data were anonymized, ensuring that all information is exclusively utilized for the analysis and objectives of this study.

Sample Size Calculation: This study employs the sample size estimation method proposed by M. Kendall et al., which recommends that the sample size for multivariate analysis should be at least 15 times the number of independent variables. In this survey, there are a total of 10 independent variables, thus according to this method, we require a minimum of 150 participants (10 independent variables multiplied by 15). To account for potential non-response or invalid questionnaires, we have increased the sample size by 20%, which is calculated as 150 multiplied by 1.20, resulting in a total sample size of 180. Consequently, the aim of this study is to include at least 180 primary caregivers of pediatric TBI patients to ensure the accuracy and reliability of the data analysis.

The target population of this study consists of primary caregivers of pediatric patients with TBI who received treatment at our hospital between February 1, 2024, and August 31, 2024. To uphold the integrity and precision of our research, stringent inclusion and exclusion criteria were established for participant selection. Inclusion criteria were as follows: children diagnosed with TBI were eligible if their Glasgow Coma Scale (GCS) score was above 9, signifying a moderate to severe level of consciousness impairment. The primary caregivers had to be at least 18 years of age, providing care for a longer duration than other caregivers, which underscored their central role in the child’s daily care. Additionally, caregivers needed to demonstrate good communication and comprehension skills and were required to voluntarily consent to participate in the study. These criteria ensured that the sample was both representative and homogeneous, aligning with the study’s objectives.

For the exclusion criteria, primary caregivers were not included if they received compensation for their caregiving services, as the study aimed to focus on non-compensated care. Caregivers with significant language or communication barriers were also excluded to ensure the accuracy of data collection and analysis. Furthermore, any primary caregiver who was unwilling to participate in the survey was respectfully not included, to uphold the principle of voluntary participation. The application of these exclusion criteria further refined the study sample, ensuring that the data collected was from a cohort that met the study’s specific requirements and thus enhancing the validity of our research findings.

In this study, we collected basic information from primary caregivers of pediatric patients with TBI, including age, gender, educational level, ethnicity, marital status, place of residence, and monthly per capita family income. These data provide a comprehensive framework of background information, aiding in understanding the caregivers’ basic situation.

Furthermore, to assess the current tatus of social support among primary caregivers of pediatric TBI patients, we utilized the Social Support Rating Scale (SSRS). Validated and reported by previous researchers [13, 14], this scale consists of 10 items across three dimensions: subjective support (4 items), objective support (3 items), and utilization of social support (3 items). The scoring method for the scale is as follows: items 1 to 4 and 8 to 10 use a four-option A, B, C, D scale, with each item scored from 1 to 4; item 5 employs a 4-point Likert scale, also scored from 1 to 4; items 7 and 9 are multiple-choice questions, with a score of 0 for selecting “none” and 1 point for each option selected under “the following sources.” The total score ranges from 12 to 66, categorized into low, medium, and high levels, with low levels scoring 22 or below, medium levels scoring between 23 and 44, and high levels scoring between 45 and 66. An elevated score on the SSRS corresponds to a greater degree of perceived social support. The scale demonstrated robust reliability and validity, as evidenced by a Cronbach’s α coefficient of 0.89, with item consistency values ranging between 0.89 and 0.94 [15, 16], These metrics affirm the SSRS’s suitability for evaluating the social support levels among primary caregivers of children with TBI, ensuring that the assessment accurately reflects the construct of interest.

After obtaining consent and support from the nursing department and the head nurses of the respective units, the researcher selected eligible subjects according to established criteria using the unit’s medical record system and verified their information. Subsequently, the researcher clearly explained the purpose of the survey to potential participants and guided them through the necessary considerations for completing the scales, ensuring their comprehensive understanding of the survey content. During the survey process, we adhered to the principles of voluntariness, anonymity, confidentiality, and informed consent, explicitly informing participants that their responses were not subject to judgment of right or wrong to alleviate their psychological pressure and concerns. Any questions that arose during the questionnaire completion were addressed by the researcher on the spot to ensure the accuracy and completeness of the data. Once the questionnaires were completed, the researcher immediately collected them and checked each one to ensure there were no omissions or errors. Finally, the collected questionnaires were numbered sequentially for subsequent data analysis and processing.

This study utilized SPSS 24.0 statistical software for data analysis. Initially, we conducted descriptive statistical analysis, employing percentages, frequencies, means, and standard deviations to characterize the general demographic features of primary caregivers and their levels of social support. We utilized the Kolmogorov-Smirnov test to assess the normality of our data distribution. This test evaluated the deviation of the data from a normal distribution by examining parameters such as the p-value. A p-value less than 0.05 indicated that the distribution significantly deviated from normality, suggesting the use of non-parametric tests. This step was essential as it guided our selection of appropriate statistical tools and ensured that our analysis was consistent with the underlying assumptions of these tools. Subsequently, we employed independent samples t-tests and analysis of variance (ANOVA) to explore differences in social support levels among primary caregivers with varying demographic characteristics. Additionally, we used Pearson correlation analysis to examine the correlation between social support and the characteristics of the primary caregivers. We acknowledged the significant influence that outliers could exert on our data analysis, potentially biasing the results and yielding misleading conclusions. Consequently, we implemented a multifaceted approach to identify and manage outliers. Our initial strategy involved a visual examination of the data through the use of box plots, density traces, and normal probability plots. These graphical tools were selected over histograms for their enhanced reliability in assessing normality, particularly when dealing with smaller sample sizes, thus providing a more accurate depiction of the data distribution and the presence of outliers. Finally, logistic regression analysis was conducted to identify factors influencing the level of social support. In all statistical tests, a p-value less than 0.05 was considered to indicate statistically significant differences.

## Results

A total of 242 main caregivers of children with TBI were included in this survey. In this study, the data were found to conform to a normal distribution. As shown in Table 1, the mean age of the 242 surveyed primary caregivers was 36.28 ± 10.13 years, with the majority being female and possessing a university education. The average monthly per capita family income was 4285.28 ± 866.95 Yuan.

**Table 1:** The characteristics of surveyed main caregivers of children with traumatic brain injury (*n* = 242)

Characteristic	Cases	Social support score	t/F	*p*
Age(y)			5.236	0.028
< 30	75	36.04 ± 10.24		
30 ∼ 40	121	38.66 ± 9.85		
> 40	46	39.28 ± 10.12		
Gender			5.381	0.095
Female	204	38.64 ± 10.27		
Male	38	38.15 ± 9.75		
Educational level			5.232	0.010
Primary school	20	34.18 ± 10.84		
Junior high school	37	36.09 ± 9.35		
Senior high school	69	38.57 ± 10.44		
University	116	39.69 ± 9.27		
Ethnicity			4.270	0.102
Han Chinese	219	38.70 ± 9.58		
Ethnic minority	23	38.34 ± 10.26		
Marital status			6.043	0.075
Divorced	25	37.19 ± 9.32		
Not divorced	217	38.74 ± 10.14		
Residence			5.237	0.036
Urban	157	39.66 ± 9.18		
Rural	85	36.78 ± 10.35		
Monthly per capita family income (Yuan)			6.445	0.003
< 3000	29	33.85 ± 10.28		
3000 ∼ 5000	140	37.90 ± 9.64		
5001 ∼ 10,000	48	39.15 ± 10.08		
> 10,000	25	41.28 ± 9.76		

As shown in Table 2, the average total score of social s of surveyed main caregivers of children with TBI was (38.52 ± 9.29), indicating that they have moderate level of social support. The primary caregivers scored highest in subjective support, followed by objective support, with the lowest scores in the utilization of support. As indicated in Table 1, there were statistical differences in the social support score of caregivers with different age, educational level, residence and monthly per capita family income (all *p* < 0.05).

**Table 2:** The social support score of surveyed main caregivers of children with traumatic brain injury (*n* = 242)

Dimensions	Total score	Average score of dimensions
Objective support	9.45 ± 3.90	3.14 ± 1.52
Subjective support	22.06 ± 5.13	5.43 ± 1.69
Utilization of support	7.82 ± 2.01	2.55 ± 0.74
Total score of social support	38.52 ± 9.29	

Upon thorough examination, our study did not identify any outliers within the dataset. As indicated in Table 3, the results of correlation analysis showed that age(*r*=-0.564), educational level(*r* = 0.602), residence(*r* = 0.542) and monthly per capita family income(*r* = 0.633) were all correlated with social support score of main caregivers (all *p* < 0.05).

**Table 3:** Pearson correlation analysis on the characteristics and social support score of main caregivers of children with traumatic brain injury

Characteristic	*r*	*p*
Age(y)	-0.564	0.040
Gender	0.114	0.109
Educational level	0.602	0.005
Ethnicity	0.120	0.125
Marital Status	0.147	0.083
Residence	0.542	0.035
Monthly per capita family income (Yuan)	0.633	0.001

As indicated in Table 4, Logistic regression analysis showed that age (OR = 2.184, 95%CI: 1.904 ∼ 3.022), educational level(OR = 2.462, 95%CI: 1.755 ∼ 4.086), residence(OR = 2.189, 95%CI: 1.805 ∼ 2.753) and monthly per capita family income(OR = 3.428, 95%CI: 2.402 ∼ 4.216) were the influencing factors of social support of main caregivers of children with TBI (all *p* < 0.05).

**Table 4:** Logistic regression analysis on the influencing factors of social support of main caregivers of children with traumatic brain injury

Variables	β	Wald	OR	95%CI	*p*
Age	0.334	0.107	2.184	1.904 ∼ 3.022	0.017
Educational level	0.308	0.114	2.462	1.755 ∼ 4.086	0.021
Residence	0.137	0.102	2.189	1.805 ∼ 2.753	0.044
Monthly per capita family income (Yuan)	0.341	0.236	3.428	2.402 ∼ 4.216	0.002

## Discussion

TBI often presents with unstable conditions, and patients are prone to various complications in the later stages, which places a significant psychological burden on family members [17]. They frequently experience fatigue and unease, and may develop psychological emotions such as anxiety and depression, as well as somatic symptoms and disturbances in eating and sleeping, severely affecting their family life [18, 19]. Even if the primary caregivers take a break from caring for the TBI child, these maladaptive coping behaviors persist [20]. Moreover, continuous stress and mental tension increase the risk of cardiovascular diseases among caregivers, and limitations in social and leisure activities may also lead to negative emotions such as social alienation and isolation [21, 22]. Previous studies [23–25] have indicated that the stress burdens felt by the families of TBI children mainly include: the child’s condition, changes in occupational status, shifts in family roles, the burden of medical expenses, loss of the main economic source, increased household labor, and potential communication barriers with the child. Therefore, social support is of great significance for the primary caregivers of TBI children. The results of this survey show that the level of social support among primary caregivers of TBI children is moderately low, which may be related to the limited and difficult access to professional social support. Additionally, the immense pressure of caregiving tasks and the irregularity of life while caring for the child may also contribute to this phenomenon.

The results of this study indicate that subjective social support scores were the highest, followed by objective social support, while the utilization of social support scored the lowest. Subjective social support contributes to the full development of an individual’s mental capacity and encourages them to actively face life’s challenges. In this survey, primary caregivers scored higher on the dimension of subjective support, which may be related to the strong family values in China [26]. When a child is hospitalized, frequent visits from relatives bring confidence and warmth to both the caregivers and the patients, allowing them to genuinely feel cared for and thus enhancing their level of social support [27, 28]. Furthermore, in this study, a higher proportion of female caregivers were observed, and women, generally more emotive than men, tend to confide in others or seek help when facing difficulties [29]. This could be one of the reasons they scored higher on the subjective support dimension. The essence of social support lies in providing caregivers with disease-related information and nursing skills to increase their understanding of the disease. Therefore, in clinical practice, various measures should be taken to expand the channels through which primary caregivers can access social support and to emphasize the importance of utilizing social support [30, 31]. This could enhance the caregivers’ sense of self-efficacy and their ability to cope with caregiving stress, ultimately improving their quality of life.

Our research findings indicate that on the variable of age, primary caregivers over the age of 40 receive higher social support scores. This phenomenon may stem from the broader social circles and richer networking resources possessed by middle-aged individuals. They are able to garner more support and assistance from social groups, organizations, and their workplace, enabling them to more effectively utilize societal resources and significantly alleviate the various burdens they bear [32, 33]. In contrast, younger primary caregivers often lack a stable support system and face greater workplace and economic pressures, leading to a relative scarcity of available social support resources [34, 35]. Therefore, healthcare professionals in clinical settings should pay special attention to younger primary caregivers of TBI patients, providing them with increased support and assistance to reduce their burdens and enhance their quality of life [36]. Furthermore, differentiated support strategies can be implemented for primary caregivers of different age groups. For instance, young caregivers could be offered career guidance and financial assistance to help establish a stable support system, while middle-aged caregivers might benefit from health management consultations and community resource connections to better utilize existing social resources [37, 38]. Through such measures, the social support levels of all primary caregivers can be more effectively elevated, promoting their overall physical and mental well-being.

The level of cultural knowledge among primary caregivers is one of the significant influencing factors of their socioeconomic status. Typically, caregivers with higher levels of cultural knowledge are better equipped to meet the demands of modern society, securing more employment opportunities and exhibiting greater potential for career advancement, which is directly correlated with their income levels. A solid economic foundation not only provides them with more material resources but also affords them the time and energy to engage in social networking platforms [39, 40]. Through these platforms, they can establish connections with a broader social demographic, gaining access to more information and assistance, thereby acquiring richer social support. Furthermore, the place of residence significantly impacts the economic income and level of social support available to primary caregivers. Caregivers residing in urban areas often benefit from a better employment environment and higher salaries, which not only elevates their quality of life but also provides them with more opportunities to participate in social activities, thus building more comprehensive social support networks. In contrast, caregivers in rural areas may face greater economic and social challenges due to limited resources and restricted access to information [41]. It is also noteworthy that the level of a family’s monthly income is a crucial factor affecting the level of social support. Families with higher monthly incomes are often able to allocate more resources to meet the diverse needs of family members, including education, healthcare, and entertainment [42, 43]. These investments not only enhance the quality of life for family members but also help them establish broader social connections, leading to greater social support. This support encompasses not only material assistance but also emotional support and information exchange, playing a vital role in increasing the well-being and social adaptability of caregivers [44, 45].

Enhancing the social support for primary caregivers of pediatric patients is a complex and multidimensional task that requires the concerted efforts of families, communities, professional institutions, and governments. Firstly, family support serves as the foundation, with family members actively participating in the care of pediatric patients, sharing responsibilities, and regularly convening family meetings to discuss the needs of the patients and the stressors faced by the caregivers. Secondly, linking to community resources, such as community centers, charitable organizations, and support groups, is essential for establishing a community support network that facilitates information sharing and mutual assistance. Professional consultation and psychological support are also crucial, offering psychological counseling services to help caregivers manage stress and emotional issues, and regularly organizing mental health lectures and workshops [46]. Economic assistance is vital for families facing financial difficulties, providing financial aid or guidance on how to access it, along with advice on effective financial management. Education and training can enhance the capabilities of caregivers by offering education and training on pediatric conditions and caregiving skills, thereby increasing their understanding of the patients’ conditions and their caregiving abilities [47]. Establishing support groups provides a platform for caregivers to share experiences, emotions, and advice, with support group meetings available through both online and offline channels. Leveraging social media and online platforms can help establish a community for pediatric caregivers, offering information, resources, and emotional support online [48]. Health education and advocacy can raise public awareness of the needs of pediatric patients and caregivers, promoting the importance of social support for pediatric caregivers through media and public events. Interprofessional team collaboration can provide comprehensive support by establishing a team of health and social service professionals, including physicians, nurses, social workers, psychotherapists, and other relevant specialists [49]. Lastly, personalized support plans can meet the specific needs of each caregiver by devising tailored support plans for each individual.

The present study, while rigorous in its approach, carries certain limitations that could influence the generalizability and precision of its findings. Firstly, the single-center, cross-sectional methodology, though it met the required sample size, may have introduced biases related to specific demographic and geographic characteristics due to the limited number of participants. This constraint could affect the representativeness of the sample and, by extension, the applicability of the results to other populations. Furthermore, our assessment of the social support levels among primary caregivers was limited to a select set of variables, which may have overlooked other significant factors that could impact social support. This selective focus could potentially lead to an incomplete understanding of the multifaceted nature of social support in this context. In light of these considerations, we recommend that future research endeavors aim for a larger and more diverse sample size to capture a wider range of demographic and geographic diversity. Additionally, broadening the scope of variables under investigation would allow for a more comprehensive analysis of the various factors that may influence the social support levels of caregivers.

## Conclusion

In summary, the findings of this survey indicate that the primary caregivers of pediatric TBI patients have a moderate level of social support, suggesting a significant potential for improvement. The social support levels of caregivers are significantly influenced by a constellation of factors, including age, educational background, residential area, and household economic status. These findings underscore the importance of developing and implementing interventions in clinical practice that are specifically tailored to address these pivotal determinants. By doing so, we can effectively bolster the social support levels of primary caregivers of pediatric patients with TBI, thereby potentially improving their overall well-being and care capacity. This targeted approach is essential for optimizing the support systems available to these caregivers and enhancing the quality of care they can provide to their children.

## Data Availability

The data associated with the paper are not publicly available but are available from the corresponding author on reasonable request.

## Abbreviations

TBI: Traumatic Brain Injury
GCS: Glasgow Coma Scale
SSRS: Social Support Rating Scale
